# Romiplostim-N01 for cancer therapy-induced thrombocytopenia: A propensity score-matched cohort study

**DOI:** 10.1097/MD.0000000000048855

**Published:** 2026-05-29

**Authors:** Ting Yu, Zhiman Xiong, Yongfeng Su, Yan Jian, Longyan Liu, Yaqi Wang

**Affiliations:** a Department of Medical Oncology, Jiangxi Cancer Hospital, The Second Affiliated Hospital of Nanchang Medical College, Jiangxi Clinical Research Center for Cancer, Nanchang, Jiangxi, China; b Department of Radiation Oncology, Jiangxi Cancer Hospital, The Second Affiliated Hospital of Nanchang Medical College, Jiangxi Clinical Research Center for Cancer, Nanchang, Jiangxi, China; c Jiangxi Medical College, Nanchang University, Nanchang, China; d Department of Medical Laboratory, Jiangxi Cancer Hospital, The Second Affiliated Hospital of Nanchang Medical College, Jiangxi Clinical Research Center for Cancer, Nanchang, China.

**Keywords:** cancer therapy-induced thrombocytopenia, recombinant human interleukin-11, recombinant human thrombopoietin, romiplostim-N01, solid tumors

## Abstract

Cancer therapy-induced thrombocytopenia (CTIT) is a common complication in patients with solid tumors during their cancer treatment. The sole therapeutic agents approved for this indication are recombinant human interleukin-11 (rhIL-11) or recombinant human thrombopoietin (rhTPO). Romiplostim-N01 is a novel thrombopoietic agent launched in China in 2024, which has demonstrated preliminary therapeutic efficacy in the management of CTIT. This retrospective study compared romiplostim-N01 with rhIL-11 or rhTPO for CTIT management. Ninety-two matched consecutive patients with grade ≥2 CTIT (platelet [PLT] count < 75 × 10^9^/L) were grouped into either the romiplostim-N01 group or the rhIL-11/rhTPO group. The primary endpoint was the proportion of treatment-marked responders, defined as patients who reached a PLT count of ≥100 × 10^9^/L and at least 30 × 10^9^/L higher than the pretreatment baseline within 7 days of treatment. Propensity score matching and a multivariate logistic regression model were used to estimate the treatment effects of the 2 drugs on CTIT. The 7-day marked response rate was significantly higher in the romiplostim-N01 group compared with the control group (58.7% vs 28.3%, *P* < .05), whereas the 14-day overall response rate was comparable between the 2 groups (89.1% vs 89.1%, *P* > .05). The median duration of chemotherapy delay was significantly shorter in the romiplostim-N01 group compared with the rhIL-11/rhTPO group (5.5 vs 9.5 days, *P* < .001). Multivariate regression analysis confirmed that romiplostim-N01 was independently associated with an elevation in PLT count. These findings position romiplostim-N01 as a favorable alternative to rhIL-11 or rhTPO in CTIT, given its enhanced early PLT response and reduced risk of chemotherapy disruption.

## 1. Introduction

Cancer therapy-induced thrombocytopenia (CTIT) refers to thrombocytopenia in cancer patients caused by anticancer treatments, including chemotherapy, radiotherapy, targeted therapy, and immunotherapy.^[1]^ As a prevalent adverse effect of antineoplastic therapy, CTIT triggers chemotherapy dose reductions or delays, and elevates bleeding risk, transfusion demand, and even life-threatening hemorrhage from vital organs.^[2,3]^ It also prolongs hospitalization and increases monitoring costs, adding a substantial economic burden. Effective CTIT management is thus pivotal for optimizing oncological outcomes and quality of life.

Currently, platelet (PLT) transfusion remains the standard for severe CTIT, yet it is constrained by blood scarcity, infectious transmission risk, and alloantibody induction leading to transfusion refractoriness. In immune checkpoint inhibitor (ICI)-associated immune thrombocytopenia (ITP), transfusion may further augment thrombosis risk.^[1]^ Beyond PLT transfusion, the primary agents approved for the indication of CTIT are recombinant human interleukin-11 (rhIL-11) and recombinant human thrombopoietin (rhTPO).^[1]^ However, rhIL-11 carries cardiac toxicity that requires close monitoring and cautious use in patients with heart disease or dose adjustment in those with renal impairment (creatinine clearance < 30 mL/min).^[1,4]^ rhTPO shares 99% homology with endogenous TPO, risking neutralizing antibody induction that may lead to treatment failure or refractory thrombocytopenia, while overuse may cause thrombocytosis and thrombosis.^[1,5]^ These limitations underscore the ongoing difficulties in CTIT management and highlight the pressing need for new and improved treatment options in this area.

In recent years, thrombopoietin receptor agonists (TPO-RAs) have emerged as promising candidates for CTIT.^[6]^ Though not yet approved for this indication, romiplostim is a prototypical TPO-RA that binds native TPO receptors to robustly activate megakaryocyte proliferation, differentiation, and maturation. Critically, its lack of homology with endogenous TPO eliminates neutralizing antibody risk.^[7]^ A Phase II study showed that 93% of patients with advanced solid tumors and chemotherapy-induced thrombocytopenia (CIT, a subset of CTIT) achieved PLT normalization within 3 weeks of romiplostim; control patients crossing over to romiplostim also had steady PLT elevation.^[8]^ In April 2024, romiplostim-N01 (China’s first romiplostim biosimilar, NMPA-approved) demonstrated substantial efficacy in adult ITP, with 61.8% achieving sustained responses at week 24 in its pivotal Phase III study.^[9]^ Two registrational Phase III trials (NCT05554913, NCT05851027) for CTIT are ongoing.

This study investigates the preliminary efficacy and safety of romiplostim-N01 versus rhIL-11 and rhTPO in moderate-to-severe CTIT, aiming to provide evidence-based support for CTIT management in China.

## 2. Materials and methods

### 2.1. Study subjects

This retrospective study was conducted at the Jiangxi Provincial Cancer Hospital. Patients with solid tumors diagnosed as CTIT between June 2024 and August 2025 were included in this study. The study received approval from the Medical Ethics Committee of Jiangxi Cancer Hospital (Approval No.: 2024ky164).

Patients meeting the following criteria were included in this study: aged ≥ 18 years with pathologically confirmed solid malignancies; diagnosed with CTIT (PLT count < 75 × 10^9^/L) due to receiving antitumor treatment such as chemotherapy, radiotherapy, targeted therapy, and immunotherapy; and planned to continue antineoplastic therapy. Patients were excluded if they satisfied any of the following criteria: patients with hematological malignancies; presence of other diseases that could cause thrombocytopenia, such as aplastic anemia, chronic liver disease, hypersplenism, and ITP; complicated with severe infection; bone marrow involvement by solid tumors; dysfunction of major organs; and expected survival time <3 months.

### 2.2. Methods

Patients were divided into the study group and the control group based on different treatment regimens administered after the clinical onset of CTIT. The study group received romiplostim-N01 (Qilu Pharmaceutical Co., Ltd.), which was administered at a fixed dose of 250 μg, once weekly, via subcutaneous injection. The control group received either rhIL-11 (Qilu Pharmaceutical Co., Ltd.) at 3 mg per day via subcutaneous injection or rhTPO (Shenyang Sansheng Pharmaceutical Co., Ltd.) at 15,000 units per day via subcutaneous injection. Thrombopoietic agent administration was discontinued when the PLT count met either of the following criteria: an increase of ≥50 × 10^9^/L above baseline or an absolute PLT count of ≥100 × 10^9^/L.

### 2.3. Definitions of endpoints

Patients underwent assessments of their PLT counts every 2 to 3 days. The primary endpoint was the proportion of patients who achieved a marked PLT response, defined as patients who reached a PLT count of ≥100 × 10^9^/L and at least 30 × 10^9^/L higher than the pretreatment baseline within 7 days of treatment. Secondary endpoints included the proportion of patients who achieved an overall PLT response (marked PLT response + good PLT response) in each group within 14 days of initiating study treatment; the median delay duration (defined as the last chemotherapy cycle to the resumption of the scheduled chemotherapy, minus the planned duration of the antitumor treatment cycles); the PLT transfusion rate; and safety. Efficacy was graded based on PLT count changes and bleeding status, defined as follows:

- Marked response: PLT count ≥100 × 10^9^/L and at least 30 × 10^9^/L higher than the pretreatment baseline within 7 days of treatment.
- Partial response: PLT count ≥100 × 10^9^/L and at least 30 × 10^9^/L higher than the pretreatment baseline within 14 days of treatment.
- Minor response: PLT count ≥100 × 10^9^/L or at least 30 × 10^9^/L higher than the pretreatment baseline within 14 days of treatment, with no bleeding symptoms.
- No response: PLT count increase of <30 × 10^9^/L from the pretreatment baseline within 14 days, no change, decrease, or presence of bleeding symptoms.

Efficacy rates were calculated as:


$$\begin{array}{cc} & Marked\;response\;rate\\ & =(number\;of\;patients\;with\;marked\;response\\ & /total\;patients)\times 100\%. \end{array}$$

$$\begin{array}{cc} & Marked\;response\;rate\\ & =(sum\;of\;patients\;with\;marked\;and\;partial\;responses\\ & /total\;patients)\times 100\%. \end{array}$$

### 2.4. Statistical analysis

Before formal analyses, variables with more than 25% missing values were deleted. Additionally, to reduce bias resulting from missing variables, we used multiple imputation by chained equations to address the missing data. Descriptive statistics were used to analyze the data. Categorical variables were presented as counts and percentages. The distribution of continuous variables was tested for normality using the Shapiro–Wilk test. Normally distributed data were presented as mean ± standard deviation and analyzed by an independent *t* test or a corrected *t* test; non-normally distributed data were expressed as median (interquartile range) and analyzed by the Mann–Whitney *U* test. Categorical variables were compared using the χ^2^ test or the Fisher exact test. Logistic regression was applied for univariate and multivariate analyses.

To minimize the impacts of potential confounders and selection bias, propensity score matching (PSM) was used to compensate for differences in baseline patient characteristics between groups. A propensity score was calculated using logistic regression, and 1:1 patient matching was performed using the nearest-neighbor matching method without replacement. Variables included age, sex, body mass index, cancer type, stage, antitumor therapy regimen, whether combined with radiotherapy (yes or no), previous antitumor treatment cycles, history of pelvic radiotherapy (yes or no), baseline PLT count, and presence of grade ≥4 leukopenia (yes or no). A caliper radius equal to a standard deviation of 0.2 was set to prevent poor matching. Standardized differences were estimated before and after matching to evaluate balance, and a value of <0.05 indicated balance between groups. The aforementioned statistical analyses were performed using R, version 4.5.0 (R Foundation for Statistical Computing). A two-sided *P* value of <.05 was considered statistically significant.

## 3. Results

### 3.1. Patient characteristics

**Figure 1.**
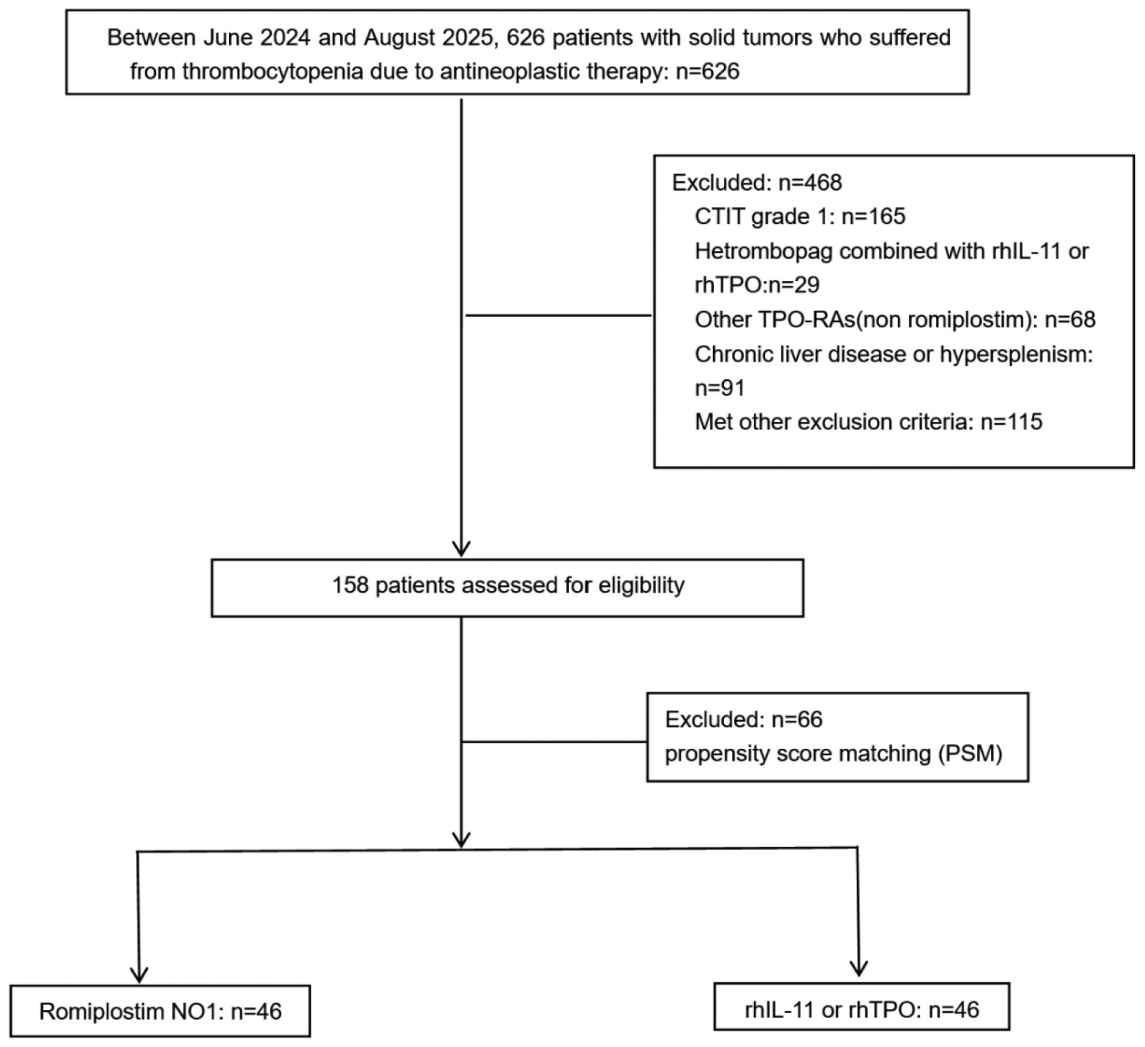
Flowchart of study subject selection for patients with solid tumors and CTIT. CTIT = cancer therapy-induced thrombocytopenia, rhIL-11 = recombinant human interleukin-11, rhTPO = recombinant human thrombopoietin, TPO-RAs = thrombopoietin receptor agonists.

Between June 2024 and August 2025, 157 patients who met the eligibility criteria were included in this study (Fig. 1). Before propensity matching, patients in the romiplostim-N01 group had significantly lower PLT counts (52.0 vs 60.0 × 10^9^/L, *P* = .009) at baseline than those in the rhIL-11/rhTPO group. Furthermore, 28.6% of patients in the rhIL-11/rhTPO group had a body mass index < 18.5, compared with 11.7% in the romiplostim-N01 group (*P* = .004). After PSM with 46 patients in each group, there were no significant differences between groups in baseline characteristics (Table 1).

**Table 1:** Baseline clinical characteristics.

Characteristic	Unmatched			Matched		
	Romiplostim-N01 (n = 63)	rhIL-11/rhTPO (n = 94)	*P* value	Romiplostim-N01 (n = 46)	rhIL-11/rhTPO (n = 46)	*P* value
Median age, yr (IQR)	57.5 (51.0, 63.0)	61.0 (51.0, 65.0)	.322	56.5 (51.0, 63.5)	60.5 (52.3, 66.5)	.377
Gender, n (%)			.494			.833
Male	50 (53.19)	37 (58.73)		26 (56.52)	27 (58.70)	
Female	44 (46.81)	26 (41.27)		20 (43.48)	19 (41.30)	
BMI, n (%)			.004			.969
<18.5	11 (11.70)	18 (28.57)		11 (23.91)	11 (23.91)	
18.5–23.9	64 (68.09)	27 (42.86)		22 (47.83)	23 (50.00)	
≥24.0	19 (20.21)	18 (28.57)		13 (28.26)	12 (26.09)	
Cancer type, n (%)			.079			.978
Lung cancer	10 (10.64)	13 (20.63)		9 (19.57)	12 (26.09)	
Ovarian cancer	6 (6.38)	3 (4.76)		2 (4.35)	2 (4.35)	
Colorectal cancer	22 (23.40)	9 (14.29)		8 (17.39)	8 (17.39)	
Gastric cancer	16 (17.02)	7 (11.11)		3 (6.52)	5 (10.87)	
Nasopharyngeal carcinoma	11 (11.70)	11 (17.46)		10 (21.74)	8 (17.39)	
Breast cancer	3 (3.19)	8 (12.70)		3 (6.52)	3 (6.52)	
Cervical cancer	10 (10.64)	3 (4.76)		4 (8.70)	3 (6.52)	
Others	16 (17.02)	9 (14.29)		7 (15.22)	5 (10.87)	
Tumor stage, n (%)			.441			.571
I–II	11 (11.70)	4 (6.35)		4 (8.70)	4 (8.70)	
III	16 (17.02)	9 (14.29)		3 (6.52)	6 (13.04)	
IV	67 (71.28)	50 (79.37)		39 (84.78)	36 (78.26)	
Antitumor therapy regimen, n (%)			.623			.966
Chemotherapy alone	40 (42.55)	22 (34.92)		16 (34.78)	17 (36.96)	
Chemotherapy + immunotherapy	27 (28.72)	17 (26.98)		12 (26.09)	13 (28.26)	
Chemotherapy + targeted therapy	6 (6.38)	8 (12.70)		3 (6.52)	4 (8.70)	
Chemotherapy + anti-angiogenic therapy	12 (12.77)	8 (12.70)		7 (15.22)	6 (13.04)	
Others	9 (9.57)	8 (12.70)		8 (17.39)	6 (13.04)	
Combined with radiotherapy, n (%)			.492			.328
No	75 (79.79)	53 (84.13)		33 (71.74)	37 (80.43)	
Yes	19 (20.21)	10 (15.87)		13 (28.26)	9 (19.57)	
Median previous antitumor treatment cycles (IQR)	5.0 (3.00, 10.0)	7.0 (4.00, 15.0)	.084	6.0 (3.0, 15.0)	7.0 (3.0, 12.8)	.793
History of pelvic radiotherapy, n (%)			.609			.765
No	78 (82.98)	55 (87.30)		39 (84.78)	40 (86.96)	
Yes	16 (17.02)	8 (12.70)		7 (15.22)	6 (13.04)	
Baseline platelet count, 10^9^/L (IQR)	52.0 (39.5, 64.5)	60.0 (51.3, 65.8)	.009	56.5 (42.8, 62.0)	52.5 (46.3, 64.5)	.981
Presence of grade ≥ 4 leukopenia, n (%)			.493			.440
No	80 (85.11)	51 (80.95)		35 (76.09)	38 (82.61)	
Yes	14 (14.89)	12 (19.05)		11 (23.91)	8 (17.39)	

BMI = body mass index, IQR = interquartile range, rhIL-11 = recombinant human interleukin-11, rhTPO = recombinant human thrombopoietin.

* Two sample *t* test; Pearson chi-squared test; Wilcoxon rank sum test; Fisher exact test.

### 3.2. Primary endpoint

Before matching, the romiplostim-N01 group exhibited a higher proportion of patients who achieved a marked response (55.6%, 35/63) compared with the rhIL-11/rhTPO group (28.7%, 27/94), with a difference in proportion of 30.4% (95% confidence interval [CI], 11.2–49.7). After PSM, the romiplostim-N01 group had a higher proportion of patients who achieved a marked response (58.7% vs 28.3%, *P* = .003) compared with the rhIL-11/rhTPO group (Table 2).

**Table 2:** The rate of platelet response before PSM and after PSM.

Response	Unmatched			Matched		
	Romiplostim-N01 (n = 63)	rhIL-11/rhTPO (n = 97)	*P* value	Romiplostim-N01 (n = 46)	rhIL-11/rhTPO (n = 46)	*P* value
Marked response, % (95% CI)	55.6 (42.5–68.1)	28.7 (19.9–39.0)	<.001	58.7 (43.2–73.0)	28.3 (16.0–43.5)	.003
Overall response, % (95% CI)	87.3 (76.5–94.4)	86.2 (77.5–92.4)	.838	89.1 (76.4–96.4)	89.1 (76.4–96.4)	>.99
Marked response, n (%)	35 (55.6)	27 (28.7)		27 (58.7)	13 (28.3)	
Partial response, n (%)	20 (31.8)	54 (57.5)		14 (30.4)	28 (60.9)	
Minor response, n (%)	4 (6.35)	6 (6.4)		2 (4.4)	3 (6.5)	
No response, n (%)	4 (6.35)	7 (7.5)		3 (6.5)	2 (4.4)	
Antitumor therapy delay, n (%)			.005			.05
Yes	51 (81.0%)	90 (95.7%)		37 (80.4%)	44 (95.7%)	
No	12 (19.0%)	4 (4.3%)		9 (19.6%)	2 (4.3%)	
Platelet transfusion, n (%)			.827			.485
Yes	6 (9.5%)	8 (8.5%)		3 (6.5%)	4 (13.0%)	
No	57 (90.5%)	86 (91.5%)		29 (93.5%)	28 (87.0%)	

Chi-squared test; Fisher exact test.

CI = confidence interval, PSM = propensity score matching, rhIL-11 = recombinant human interleukin-11, rhTPO = recombinant human thrombopoietin.

### 3.3. Second endpoints

As shown in Table 2, before PSM, the proportion of patients who achieved overall response was similar between the 2 groups, with overall response rates of 87.3% and 86.2% (*P* = .838). After PSM, the same result was observed. The overall response rate in both groups was 89.1%. Due to unsatisfactory PLT elevation and the development of severe thrombocytopenia (PLT < 15 × 10^9^/L), 3 patients (6.52%) in the romiplostim-N01 group and 6 patients (13.04%) in the control group received PLT transfusions. There was no statistically significant difference in the PLT transfusion rate between the 2 groups (Table 2).

**Figure 2.**
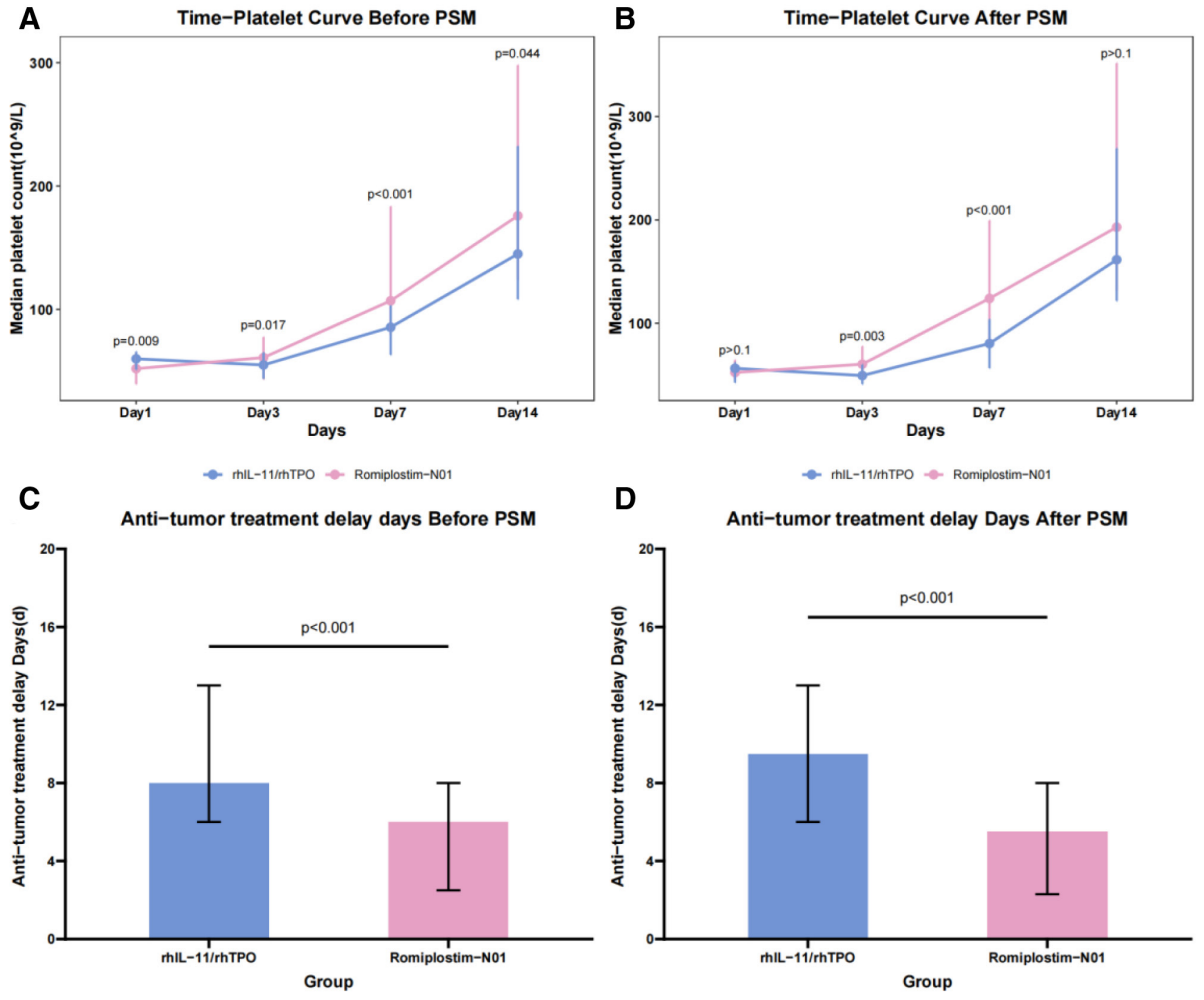
(A) The median platelet count at day 3, 7, and 14 before PSM. (B) Median platelet count at day 3, 7, and 14 after PSM. (C) The median time of antitumor treatment delays before PSM. (D) The median time of antitumor treatment delays after PSM. PSM = propensity score matching, rhIL-11 = recombinant human interleukin-11, rhTPO = recombinant human thrombopoietin.

Before PSM, the median PLT counts in the romiplostim-N01 group at 3 days, 7 days, and 14 days were 61.0 × 10^9^/L, 107.0 × 10^9^/L, and 176.0 × 10^9^/L, while in the control group, the median PLT counts were 55.0 × 10^9^/L, 85.5 × 10^9^/L, and 145.0 × 10^9^/L. After PSM, the median PLT counts at 3 days, 7 days, and 14 days in the romiplostim-N01 group and the control group, respectively, were 60.5 × 10^9^/L versus 49.5 × 10^9^/L, 124.0 × 10^9^/L versus 80.5 × 10^9^/L, and 193.0 × 10^9^/L versus 161.5 × 10^9^/L. When compared between the 2 groups, the romiplostim-N01 group showed a significantly greater and faster increase in PLT count (Fig. 2A and B).

A number of patients in both groups experienced delays in antitumor therapy. Before PSM, 51 patients (80.0%) in the romiplostim-N01 group and 90 patients (95.7%) in the control group had treatment delays. After PSM, the proportions of patients with delayed therapy were comparable, at 80.4% (37/46) in the romiplostim-N01 group and 95.7% (44/46) in the control group (*P* < .05). We also compared the median treatment delay time of the 2 groups. The results showed that before PSM, the median treatment delay times of the 2 groups were 6.0 (2.5–8.0) days and 8.0 (6.0–13.0) days, respectively. After PSM, the median treatment delay time of the romiplostim-N01 group was shortened to 5.5 (2.3–8.0) days, while that of the control group was extended to 9.5 (6.0–13.0) days (Fig. 2C and D).

### 3.4. Logistic regression

After PSM, 40 of 92 patients (46 patients in each group) achieved a marked PLT response. Using the group as the independent variable and whether a marked PLT response was achieved as the dependent variable, univariate logistic regression and multivariate logistic regression were performed. Patients in the romiplostim-N01 group had a significantly higher rate of achieving a marked PLT response than the control group (odds ratio [OR], 0.28, 95% CI, 0.12–0.66, *P* = .004). To decrease the effect of confounding factors, we further performed multivariate logistic regression. In the multivariate model, we found that a higher rate achieved marked PLT response remained in the romiplostim-N01 group. Compared with rhIL-11/rhTPO, patients who accepted romiplostim-N01 treatment were a favorable factor for a higher rate of marked response (OR, 0.26, 95% CI, 0.10–0.64, *P* = .004). However, patients who received chemotherapy combined with anti-angiogenic therapy were associated with a lower rate of marked response than those receiving chemotherapy alone (OR, 4.92, 95% CI, 1.06–22.86, *P* = .042; Table 3).

**Table 3:** Univariate and multivariate analysis of marked platelet response.

Characteristic	Univariate			Multivariate		
	OR	95% CI	*P* value	OR	95% CI	*P* value
Group						
rhIL-11/rhTPO	Ref	–		Ref	–	
Romiplostim-N01	0.28	0.12–0.66	.004	0.26	0.10–0.64	.004
Age (yr)						
<60	Ref	–				
≥60	0.88	0.38–2.01	.755			
Gender						
Male	Ref	–				
Female	0.69	0.30–1.59	.385			
BMI, n (%)						
<18.5	Ref	–				
18.5–23.9	1.37	0.49–3.81	.548			
≥24.0	1.50	0.47–4.77	.492			
Cancer type						
Lung cancer	Ref	–				
Ovarian cancer	1.85	0.16–20.94	.621			
Colorectal cancer	0.79	0.21–2.97	.729			
Gastric cancer	0.37	0.07–1.98	.245			
Nasopharyngeal carcinoma	0.97	0.27–3.53	.960			
Breast cancer	0.31	0.05–2.08	.227			
Cervical cancer	0.82	0.14–4.66	.823			
Others	0.86	0.20–3.66	.840			
Tumor stage, n (%)						
I–II	Ref	–				
III	1.25	0.19–8.44	.819			
IV	1.34	0.31–5.78	.692			
Antitumor therapy regimen, n (%)						
Chemotherapy alone	Ref	–		Ref	–	
Chemotherapy + immunotherapy	2.41	0.83–7.03	.106	2.69	0.86–8.37	.088
Chemotherapy + targeted therapy	1.81	0.35–9.41	.481	2.10	0.37–12.03	.403
Chemotherapy + anti-angiogenic therapy	4.52	1.05–19.54	.043	4.92	1.06–22.86	.042
Others	1.81	0.51–6.40	.358	1.71	0.45–6.50	.431
Combined with radiotherapy, n (%)						
No	Ref	–				
Yes	1.47	0.55–3.96	.442			
Previous antitumor treatment cycles						
<10	Ref	–				
≥10	1.04	0.45–2.44	.925			
History of pelvic radiotherapy, n (%)						
No	Ref	–				
Yes	0.88	0.27–2.86	.834			
Baseline platelet count, 10^9^/L						
<50	Ref	–				
≥50	1.02	0.43–2.42	.969			
Presence of grade ≥ 4 leukopenia, n (%)						
No	Ref	–				
Yes	1.07	0.39–2.98	.892			

BMI = body mass index, CI = confidence interval, OR = odds ratio, rhIL-11 = recombinant human interleukin-11, rhTPO = recombinant human thrombopoietin.

### 3.5. Safety

No thromboembolic or bleeding events were observed in the romiplostim-N01 group. By contrast, 3 patients in the control group experienced deep vein thrombosis, with no bleeding events reported.

## 4. Discussion

Thrombocytopenia is a common hematological adverse event (AE) during cancer treatment and a critical factor constraining treatment dose intensity. With the rapid evolution of antineoplastic therapies in recent years, thrombocytopenia is no longer restricted to traditional cytotoxic chemotherapy but also occurs commonly with precision-based therapies, including immunotherapies (e.g., PD-1/PD-L1 inhibitors, chimeric antigen receptor T-cell therapy), small-molecule targeted agents, and antibody-drug conjugates.^[1]^ Clinical data indicate a 10% to 30% incidence of CTIT across diverse treatment regimens.^[5,6]^ CTIT can lead to serious sequelae, including dose reduction, treatment discontinuation, treatment delays, and life-threatening bleeding – all of which significantly impair patients’ quality of life and long-term prognosis. Accordingly, CTIT has become a major bottleneck limiting the efficacy of multimodal cancer therapy.

Therapeutic options for CTIT remain limited in China. Currently, only 2 agents are approved for the management of CTIT: rhIL-11 and rhTPO. However, these agents are linked to relatively high rates of AEs and modest efficacy in PLT elevation, resulting in a substantial unmet clinical need.

Romiplostim, a prototypical TPO-RA, was first clinically approved in Europe and the United States in 2008. It has since expanded its indications to include chronic primary ITP and refractory aplastic anemia, with accumulating evidence supporting its utility in CIT management among patients with malignancies.^[8]^ A US retrospective study reported a 71% response rate to romiplostim in CIT: 79% of patients avoided chemotherapy dose reductions or delays, and 89% avoided PLT transfusions. Among patients with solid tumors, 85% attained normal PLT counts (≥100 × 10^9^/L) within 9 days of treatment.^[10]^ Notably, romiplostim is the only TPO-RA explicitly recommended for CIT in the NCCN Guidelines.^[11]^

In 2024, China published the *Expert Consensus on the Clinical Application of Romiplostim*, offering a novel therapeutic option for cancer patients – particularly those with hematological malignancies.^[12]^ However, romiplostim has only recently been launched in China, and data regarding its efficacy in solid tumor-associated CTIT remain scarce. This highlights the pressing need for further clinical studies to confirm its utility in this patient population.

Romiplostim exerts its pharmacological effect via binding to the extracellular domain of the TPO receptor, thereby activating multiple downstream signaling pathways (e.g., JAK2/STAT5, PI3K/Akt, ERK).^[12]^ Unlike certain non-peptide TPO-RAs, romiplostim additionally activates the STAT3 pathway, which further promotes the proliferation, differentiation, and maturation of megakaryocytes, ultimately enhancing PLT production.^[13]^ Of note, AEs requiring close monitoring (e.g., thrombosis) are comparatively rare, even with prolonged administration.^[14,15]^ Romiplostim exhibits a half-life of 1 to 4 days (median: 3.5 days) and necessitates only once-weekly administration. Consistent with this pharmacokinetic profile, our study employed a fixed dose of 250 μg (once weekly), which is consistent with the dosage form of romiplostim-N01 marketed in China (250 μg per vial).

This study is the first to compare the therapeutic efficacy of romiplostim-N01 with that of rhIL-11/rhTPO, whereas prior studies employed placebo or observation as control arms. Our findings demonstrated that the romiplostim-N01 group had significantly higher PLT levels at 7 and 14 days than the control group. Specifically, the 7-day marked PLT response rate was 58.7% in the romiplostim-N01 group, significantly exceeding the 28.3% in the control group, demonstrating more rapid and effective PLT elevation than conventional thrombopoietic agents. The 7-day marked response rate in our study was marginally lower than those reported in prior international studies.^[10,16]^ This discrepancy may be attributed to 2 key factors: first, this study enrolled CTIT patients with higher-grade thrombocytopenia (grade ≥ 2); second, there were differences in primary endpoint definitions between our study and previous research.

In terms of treatment intensity, approximately 80% of enrolled patients in this study had advanced malignancies. Most had previously received multiple cycles of antineoplastic therapy, posing considerable challenges to bone marrow reserve. Both groups had varying degrees of treatment delays or chemotherapy dose reductions attributable to grade ≥ 2 CTIT. More specifically, the median delay was 5.5 days in the romiplostim-N01 group, which was significantly shorter than the 9.5 days in the control group. This is consistent with international reports,^[17]^ further validating romiplostim-N01’s capacity to rapidly increase PLT counts and substantially reduce the risk of chemotherapy delays.

Furthermore, romiplostim-based secondary prophylaxis effectively mitigated reductions in chemotherapy dose intensity or treatment delays attributable to CIT in 70% of patients.^[18]^ During 1 year of prophylactic administration, only 1 patient (1/20) experienced deep vein thrombosis. In the present study, a 75-year-old patient with advanced rectal cancer received romiplostim-N01 for secondary prophylaxis over 7.2 months; this treatment yielded significant efficacy, with no CTIT recurrence, good tolerability, and no thromboembolic events reported.

With the widespread use of ICIs, growing evidence suggests that ICIs can induce immune-related thrombocytopenia, such as idiopathic thrombocytopenic purpura and thrombotic thrombocytopenic purpura.^[19–21]^ The underlying mechanism remains incompletely elucidated but may involve specific antibody production, aberrant cytokine secretion, and PLT surface PD-L1 expression.^[22]^ Thrombocytopenia accounts for approximately 34% of ICI-induced hematological AEs, with 23.5% of patients at risk of persistent progression.^[23]^ As reported by Liu et al,^[24]^ ICI-related thrombocytopenia is more prevalent in males, elderly individuals, and Asian populations; common indications include non-small cell lung cancer and malignant melanoma, with a median onset time of 40 to 42 days. In our study, approximately 28% of patients had previously received chemotherapy combined with ICIs, suggesting that combined immunotherapy may elevate the risk of PLT decline. Further regression analysis revealed that prior exposure to immunotherapy may be associated with a suboptimal PLT recovery response. However, the small sample size of patients with prior immunotherapy exposure and the absence of stratified analysis by immunotherapy cycle number limit the robustness of this finding, underscoring the need for further in-depth investigations.

Meanwhile, sustained angiogenesis represents a key biological hallmark of malignant tumors. Inhibiting the vascular endothelial growth factor pathway blocks tumor neovascularization and aberrant remodeling, thereby suppressing tumor proliferation, invasion, and metastasis, thus establishing anti-angiogenic therapy as a cornerstone strategy for solid tumors. The major classes of anti-angiogenic agents include monoclonal antibodies (e.g., bevacizumab, ramucirumab) and small-molecule tyrosine kinase inhibitors (e.g., lenvatinib, regorafenib, anlotinib). Beyond hypertension, proteinuria, bleeding, and thromboembolic events, anti-angiogenic agent-induced thrombocytopenia ranks second among spontaneously reported severe adverse drug reactions.^[25]^ A literature review reported that bevacizumab-induced thrombocytopenia has an approximate incidence of 10% to 13.3%, with a higher risk when combined with paclitaxel or platinum-based regimens; the underlying mechanism may involve immune-mediated peripheral PLT destruction.^[26]^ In the present study, 13 patients were enrolled who received chemotherapy plus anti-angiogenic therapy (4 with advanced lung cancer, 2 with ovarian cancer, 1 with cervical cancer, and 6 with colorectal cancer). Regimens consisted of bevacizumab plus paclitaxel ± platinum or oxaliplatin. These patients exhibited consistent clinical characteristics: more severe and prolonged thrombocytopenia, suboptimal and delayed PLT recovery, resulting in subsequent chemotherapy delays or dose reductions.

Subsequent logistic regression analysis further demonstrated that chemotherapy combined with anti-angiogenic therapy was significantly associated with a lower marked PLT response rate compared with chemotherapy alone. After adjusting for potential confounders such as baseline PLT count, bone marrow reserve, and concomitant medications, the “synergistic inhibitory effect” of the 2 therapies persisted, confirming synergistic hematological toxicity. This finding underscores the need for strengthened clinical medication safety: while maintaining antitumor efficacy, it is imperative to implement systematic management strategies to mitigate the risk of thrombocytopenia.

Importantly, the 3-drug combination regimen (chemotherapy plus immunotherapy and anti-angiogenic therapy) has emerged as a paradigm-shifting strategy in solid tumor treatment, driven by the synergistic mechanisms of “tumor cytotoxicity, tumor microenvironment remodeling, and immune activation.”^[27,28]^ However, these benefits are accompanied by heightened risks, particularly with respect to hematological toxicity. In the context of multimodal treatment, our findings demonstrated that romiplostim-N01 achieved a significantly higher marked PLT response rate than rhIL-11/rhTPO. This indicates that romiplostim-N01 was independently associated with a significantly higher OR for marked PLT elevation compared with rhIL-11/rhTPO. This efficacy advantage persisted after adjustment for confounders and holds direct clinical relevance for CTIT management, particularly in patients requiring long-term PLT support or multi-drug combination therapy. In the Chinese healthcare context, rhIL-11 has a lower direct cost but a higher incidence of AEs. Romiplostim-N01, despite a relatively higher direct cost, demonstrates superior efficacy and safety, as well as reduced PLT transfusion requirements and lower risks of chemotherapy delay, which may bring favorable comprehensive clinical and economic benefits for patients with CTIT.

This study has several limitations. As a retrospective observational study, the overall study population was heterogeneous, encompassing diverse tumor types and chemotherapy regimens. Consistent with most retrospective studies, selection bias may exist, given that analyses were restricted to patients treated with romiplostim-N01 or rhIL-11/rhTPO, without randomization. Second, this was a single-center study with a small sample size. Additionally, the follow-up duration was relatively short. Although PSM was performed to balance baseline characteristics between the 2 groups, unmeasured confounders may still exist, such as nutritional status, bone marrow reserve, hepatic and renal function, baseline inflammatory status, and severity of myelosuppression, which may potentially bias the study results. Consequently, critical questions such as whether romiplostim-N01 can improve overall survival, progression-free survival, and quality of life in cancer patients remain to be further explored. These uncertainties should therefore be addressed in future well-designed, adequately powered clinical trials.

## 5. Conclusion

In conclusion, our study preliminarily confirms that romiplostim-N01 confers a distinct advantage in rapidly elevating PLT counts among patients with CTIT, alongside a favorable safety profile. It therefore represents a promising treatment option for moderate-to-severe CTIT. To further validate its efficacy and safety, large-sample, multicenter randomized controlled trials are warranted.

## Acknowledgments

The authors thank all participating patients, their families, and physicians for collaborating in data collection.

## Author contributions

**Conceptualization:** Ting Yu, Zhiman Xiong.

**Methodology:** Yan Jian, Yaqi Wang.

**Funding acquisition:** Yaqi Wang.

**Data curation:** Ting Yu, Zhiman Xiong, Yongfeng Su, Longyan Liu.

**Formal analysis:** Ting Yu, Zhiman Xiong, Yan Jian.

**Investigation:** Ting Yu, Yongfeng Su, Longyan Liu, Yaqi Wang.

**Project administration:** Ting Yu.

**Supervision:** Ting Yu, Yongfeng Su, Yan Jian, Longyan Liu, Yaqi Wang.

**Visualization:** Ting Yu.

**Validation:** Zhiman Xiong, Yongfeng Su, Yan Jian, Longyan Liu.

**Writing – original draft:** Ting Yu, Zhiman Xiong.

**Writing – review & editing:** Yaqi Wang.
